# The Use of Hyperbaric Oxygen Therapy and Corticosteroid Therapy in Acute Acoustic Trauma: 15 Years’ Experience at the Czech Military Health Service

**DOI:** 10.3390/ijerph18094460

**Published:** 2021-04-22

**Authors:** Richard Holy, Sarka Zavazalova, Klara Prochazkova, David Kalfert, Temoore Younus, Petr Dosel, Daniel Kovar, Karla Janouskova, Boris Oniscenko, Zdenek Fik, Jaromir Astl

**Affiliations:** 1 Department of Otorhinolaryngology and Maxillofacial Surgery, Military University Hospital, 16902 Prague, Czech Republic; richard.holy@uvn.cz (R.H.); sarka.zavazalova@uvn.cz (S.Z.); tny1996@gmail.com (T.Y.); daniel.kovar@uvn.cz (D.K.); janouskova.karla@uvn.cz (K.J.); jaromir.astl@uvn.cz (J.A.); 2 Third Faculty of Medicine, Charles University, 10000 Prague, Czech Republic; klara.prochazkova@fnkv.cz; 3 Department of Otorhinolaryngology, University Hospital Kralovské Vinohrady, 10034 Prague, Czech Republic; 4 Department of Otorhinolaryngology and Head and Neck Surgery, University Hospital Motol, First Faculty of Medicine, Charles University, 15006 Prague, Czech Republic; zdenek.fik@fnmotol.cz; 5 The Institute of Aviation Medicine, 16000 Prague, Czech Republic; petrdosel@atlas.cz (P.D.); oniscenko@ulz.cz (B.O.)

**Keywords:** acute acoustic trauma, noise induced hearing loss, tinnitus, hyperbaric oxygen therapy

## Abstract

Background: Acute acoustic trauma (AAT) ranks, among others, as one common cause of inner ear function impairment, especially in terms of military personnel, who are at an increased exposure to impulse noises from firearms. Aim of this study: 1. We wanted to demonstrate whether early treatment of AAT means a higher chance for the patient to improve hearing after trauma. 2. We find the answer to the question of whether hyperbaric oxygen therapy (HBO2) has a positive effect in the treatment of AAT. Methods: We retrospectively analyzed data for the period 2004–2019 in patients with AAT. We evaluated the therapeutic success of corticosteroids and HBO2 in a cohort of patients with AAT n = 108 patients/n = 141 affected ears. Results: Hearing improvement after treatment was recorded in a total of 111 ears (79%). In terms of the data analysis we were able to ascertain, utilizing success of treatment versus timing: within 24 h following the onset of therapy in 56 (40%) ears—54 (96%) ears had improved; within seven days following the onset the therapy was used in 55 (39%) ears—41 (74%) ears had improved; after seven days the therapy started in 30 (21%) ears—16 (53%) ears had improved. Parameter latency of the beginning of the treatment of AAT was statistically significant (*p* = 0.001 and 0.017, respectively). The success of the medical protocols was apparent in both groups—group I (treated without HBO2): n = 61 ears, of which 50 (82%) improved, group II (treated with HBO2): n = 73 ears, of which 56 (77%) improved. Group II shows improvement at most frequencies (500–2000 Hz). The most serious sensorineural hearing loss after AAT was at a frequency of 6000 Hz. Conclusion: Analysis of our data shows that there is a statistically significant higher rate of improvement if AAT treatment was initiated within the first seven days after acoustic trauma. Early treatment of AAT leads to better treatment success. HBO2 is considered a rescue therapy for the treatment of AAT. According to our recommendation, it is desirable to start corticosteroid therapy immediately after acoustic trauma. If hearing does not improve during the first seven days of corticosteroid therapy, then HBO2 treatment should be initiated.

## 1. Introduction

During acute acoustic trauma (AAT), the inner ear becomes mechanically damaged, after a short-impact acoustic impulse (intensity of 90–130 dB for a duration of 1 ms).

In terms of pathology, protective middle ear reflexes are blocked, which cause an alteration of action potential formation. Vasospasm of microcirculation and hypoxia of sensory cells occur, in order to prevent metabolic imbalance. Pathological processes may result in damaging hair cells and dendrites of primary auditory neurons that consequently induce a transition stage between the regeneration and cell death. This so-called transition stage may primarily influence the line of therapy [1].

Typical AAT symptoms include high-frequency sensorineural hearing loss (4 kHz and higher, while 1–2 kHz influenced minimally) and tinnitus.

Vertigo or spontaneous nystagmus are rarely present [2].

There are different severities of AAT, for instance, less severe onset (which is less frequent) causes reversible hearing loss—temporary threshold shifts (TTS)—hearing is restored within 24 h from acoustic trauma [3,4]. More severe onset (which is more frequent) causes irreversible hearing loss—permanent threshold shifts (PTS) [3,4,5].

The optimal treatment for AAT is yet to be defined [3,4,5]. Some animal studies display that hyperbaric oxygen therapy (HBO2) combined with corticosteroid therapy improve the functional and morphological conditions of the inner ear, by allowing permanent therapeutic effect through noise-induced cochlear hypoxia [3,4,5]. However, the negative effect of HBO2 is also reported in the literature (animal study) [6]. Some papers states that expectant non-interventional recovery of hearing does not belong among therapeutic alternatives as in most cases hearing recovery is incomplete. After AAT a partial hearing loss and tinnitus usually persist. On the other hand, it is described in literature—spontaneous hearing recovery without the treatment. It can probably be attributed to a naturally occurring phenomena [4,7]. Kuznecov et al. present in their manuscript the groups of the most used drugs for the pharmacological correction of hearing loss after AAT: corticosteroids, antioxidants, nootropics, antihypoxants, and others [8].

Administration of therapy (of AAT) is quintessentially begun within 24 h after the acoustic trauma [9,10,11]. Using the data analysis of our group of patients undergoing standardized medical protocols (corticosteroid therapy, HBO2 therapy), we aim to prove a causal connection between starting early treatment and a better prognosis for hearing loss improvement, thus proving our objective [9,10,11].

Although 75% of AAT of cases can be classified as occupational accidents, an absolutely necessary precondition is good awareness and cooperation of military general practitioners, through immediate administration of corticosteroid therapy at the site of trauma with prompt referral of soldiers to the relevant military hospital for further therapy (corticosteroid therapy, vasodilator treatment, and HBO2 therapy). For instance, in Finland, just as in the Czech Republic, several hundred soldiers suffer from AAT every year despite strict security regulations dealing with shooting from firearms in defense forces, in the USA, 20–30% of soldiers experience hearing impairment [1,9,12,13,14,15].

### Issues of HBO2 Therapy in AAT from the Perspective of a Hyperbaric Medicine Expert

Hyperbaric oxygen therapy is a type of inhalation treatment using highly concentrated oxygen inside a hyperbaric chamber, in which the pressure is higher than atmospheric pressure. The therapeutic pressures range between 200–280 kPa (2–2.8 ATA = absolute technical atmosphere). The therapeutic excess pressures range between 100–180 kPa (1–1.8 ATA). The usual treatment exposure time is 120 min. Patients with hearing loss are exposed to HBO2 therapy once a day [8,9,16].

HBO2 therapy contributes to AAT treatment by improving oxygenation of the inner ear, which results in the adjustment of transmembrane potential, activation of cell metabolism, and regeneration of ionic balance. Rheologically, the effect of oxygen diffusing through the oval window leads to a decrease in hematocrit and blood viscosity [8,9,10].

Several ear disorders correlated in literature with anaerobic bacteria infection, influencing the prognosis of serious diseases such as lateral cervical and mediastinal involvements; however excellent response to hyperbaric therapy combined with antibiotics and cortisone drugs has been reported [17,18].

Aim of this study: Demonstrate whether early treatment of AAT means a higher chance of improving hearing after trauma. Answer the question of the positive effect of HBO2 in the treatment of AAT.

## 2. Materials and Methods

We retrospectively analyzed data from n = 108 patients/n = 141 damaged ears (33 patients with bilateral AAT) treated in the period between 2004 and 2019. This cohort of patients consisted of 97 men (90%) and 11 women (10%). A total of 65 representatives of group A (soldiers) (60%), 43 representatives of group B (civilians) (40%). The age range was between 18 and 82 years, the average age was 38 years.

The etiology of the AAT source was recorded: after shooting 102 (72%), after an explosion 11 (8%), after a music concert 10 (7%), and others 18 (13%) (after a barking dog, after whistling, after shouting, after car battery explosion, after incorrect fitting of earplugs/earmuffs).

### 2.1. Division into Groups According 

We divided the cohort of patients into groups:

Group A—were soldiers (sound intensity at AAT was up to 170 dB)
Group B—were civilian persons (sound intensity at AAT was up to 120 dB)

We also divided the cohort of patients into groups according to the start of treatment:

Parameter latency of the beginning of the treatment of AAT within 24 h
Parameter latency of the beginning of the treatment of AAT within 7 days
Parameter latency of the beginning of the treatment of AAT after 7 days

Further division of the patient cohort into groups was according to the method of treatment:

Group I—patients were treated with corticosteroids + vasodilatory infusion, without hyperbaric oxygen therapy. In this group, the age range was between 20 and 82 years, the average age was 33 years.
Group II—patients were treated with corticosteroids + vasodilatory infusion + hyperbaric oxygen therapy. In this group, The age range was between 18 and 69 years, the average age was 38 years.

Whilst monitoring the effect of particular medical protocols we singled out a group of patients (“Singled out group p. o. vasodilatants”— who were treated only by vasodilators p.o. (betahistin-dihydrochlorid 24 mg, vinpocetin), i.e., 7 damaged ears. In comparison with the effect of the medical protocol without HBO2 vs. with HBO2, we finally evaluated a group of 134 damaged ears. Audiometric measurements were taken with the help of pure tone audiometry (PTA) (measurement dB HL). We used the device Orbiter 992 with Headphone TDH 39 and with the valid Certificate from the Metrology Department, Czech Republic. Measurements were taken before and at the end of treatment. First PTA was performed by soldiers within 24 h after AAT. First PTA was performed by civilians as soon as they patients visit our clinic. Last PTA was performed two months after finishing therapy. 

### 2.2. Medical Protocol

On the day of AAT or on the day of the first examination—corticosteroid therapy started with Solu-Medrol (1st day: Solu-Medrol 125 mg; 2nd day: Solu-Medrol 80 mg; 3rd day: Solu-Medrol 40 mg; in 100 mL of physiological solution i.v.) in addition to vasodilation infusions (20 mg of ethyl apovincaminate alias known as “vinpocetine”; in 250 mL of physiological solution; 120 min; 10 days). In the case there was no amelioration of the condition within 7 days (verified by PTA), we decided to start hyperbaric oxygen therapy as soon as possible (10 exposures in the hyperbaric chamber in the Institute of Aviation Medicine, Prague; pressure 2.5 atmosphere for 120 min). A special group of patients (n = 7 ears) were treated only with vasodilators peroral—vinpocetine 10 mg one tablet twice per day, betahistin-dihydrochlorid 24 mg one tablet three times per day.

The standard medical protocol of HBO2 therapy used in this study includes a compression phase during which the pressure in the hyperbaric chamber is increased for a period of 15 min to the therapeutic level of 250 kPa (2.5 ATA). Both initial compression phase, from surface (1.0 ATA) to the treatment depth (250 kPa = 2.5 ATA), and the final decompression from depth to surface are in, they last 30 min together (15 min/each at a compression/deco- speed of 1 m/min). The complete ‘dive’ table used in this study took 90 min, including an interposed break of 5 min in ambient air at depth (such a break is applied as conservative prevention from hyperbaric oxygen possible side effects). During compression, the patient is ventilating atmospheric air (within the chamber), because wearing an oxygen mask would obstruct performing active maneuvers for equalizing the increase of pressure to the middle ear. Compression relates to the most frequent occurrence of baric problems (usually earache or pain of paranasal sinuses) because of insufficient ventilation function of the Eustachian tube. Final decompression lasts 15 min and there are usually no baric problems. The standard minimum number of Hyperbaric Oxygen Treatments (Tx) required in this case is 10 [2,8,9].

### 2.3. Statistical Analysis of Data

All statistical analyses were performed with IBM SPSS Statistics (version 22.0; SPSS, IBM, Armonk, NY, USA). We used the non-parametric Mann–Whitney test and Fisher’s exact test and *p* value < 0.05 was used to establish statistical significance. 

## 3. Results

Results of lateral prevalence of AAT did not show a greater difference in significance between Group A and Group B. Bilateral damage occurred in 33 patients (30%).

Hearing improved in statistically younger patients (average age 34, 4 years; level of significance *p* = 0.001) as compared to older ones (average 44, 4 years).

Table 1 shows the success rate of AAT treatment. In total, 79% improved, Group A vs. Group B, 70% vs. 81%, respectively. Hearing improvement in standard terms (when hearing loss threshold after treatment was above 20 dB HL) was 41% in total, Group A vs. Group B, 39% vs. 29%, respectively. In group B, there was a statistically significant (at *p* = 0.012) higher partial hearing improvement after AAT treatment.

Within the group of patients with improved conditions, the figures of sound intensity before the treatment, in all frequencies, were significantly lower than in non-improved, on the level of significance 0.05 resp. 0.1 (frequency 1000, 4000, 6000 Hz).

An important parameter, early treatment of AAT treatment, is shown in Table 2. Improvement of hearing—after the treatment started within 24 h was 96%, Group A 97% and Group B 95%. After the treatment started within seven days, improvement of hearing was in 74%, 79% at group A and 70% at group B. More than seven days after treatment started, improvement of hearing was in 53%, 53% in Group A and 55% in Group B. Parameter: Latency of the beginning of the treatment—whole set, Group A and Group B, this parameter of early treatment of AAT is always statistically significant (*p* ≤ 0.001 and 0.017, respectively).

The earlier AAT treatment was started, the higher were the chances of hearing improvement (*p* ≤ 0.001 and 0.017, respectively)

**Figure 1:**
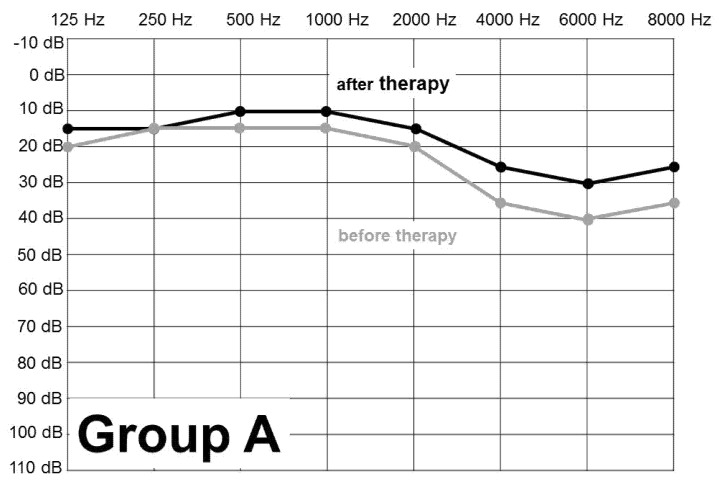
PTA before and after the treatment of AAT—Group A—soldiers (n = 83).

**Figure 2:**
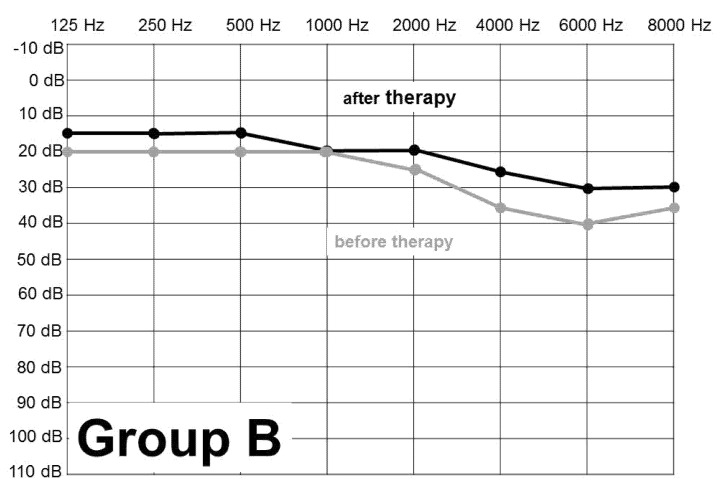
PTA before and after the treatment of AAT—Group B—civilian persons (n = 58).

**Figure 3:**
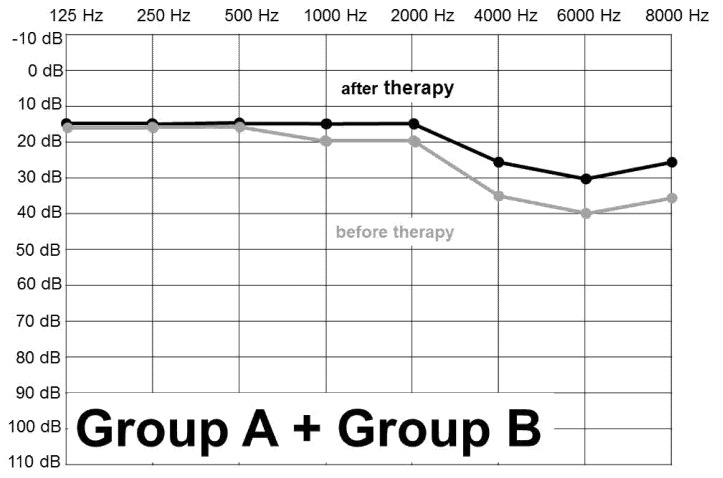
PTA before and after the treatment of AAT—in total in all AAT (n = 141 ears).

Figure 1, Figure 2 and Figure 3 show the figures of PTA curve before and after the treatment in total, in Group A and Group B. Total improvement of the hearing threshold after treatment, at the highest affected frequencies, was by 8 dB. The charts show clearly that the greatest damage of the whole set, both group A and group B, was at 6000 Hz and in descending order at 8000, 4000, 2000 Hz, respectively. 

The success of particular medical protocols with/without HBO2 is referred to in Table 3. Improvement of hearing occurred in 82% of patients from Group I (without HBO2) and 77% of patients from Group II (with HBO2). A total of 134 damaged ears were evaluated. Group “Singled out group p. o. vasodilatants” treated with peroral vasodilatants, of which seven ears were not included—see Table 3.

Table 4 shows audiometric figures before and after the treatment in total in Group I (without HBO2) and Group II (with HBO2). The whole set in Group II shows a greater amelioration at most frequencies, a larger difference in the change of sound intensity was statistically significant at 500 Hz (*p* < 0.01) and 2000 Hz (*p* < 0.05). Cases of tinnitus occurred after AAT in 58% of 141 damaged ears. In Group A, tinnitus was experienced in 63% of ears and only 52% in Group B. After treatment, tinnitus disappeared in a total of 50% of damaged ears. In Group A, tinnitus disappeared in 54% of damaged ears compared to 43% in Group B. 

Vertigo occurred in 7% of patients from the total number of 108 patients (141 ears). 

Nystagmus occurred in 3% of the total number of 108 patients. Vertigo and nystagmus disappeared after the treatment.

## 4. Discussion

Available sources state that, at present, AAT treatment consists of applying the combination of corticosteroid therapy and HBO2 therapy [1,3,4,11]. Studies proved the presence of steroid receptors in the inner ear. Steroids participate in forming ionic balance in the inner ear, stabilization of the cell membrane, and increased perfusion and inhibition of anti-inflammatory cytokines [4].

During HBO2 therapy, the patient inhales oxygen (100%) greater than at atmospheric pressure. This HBO2 therapy is generally administered at 2.0–2.8 atmospheres for a period of 60–90 min, usually once a day within 10–15 days [1,2,3]. HBO2 therapy increases the level of oxygen dissolved in the blood, which is subsequently transferred in larger amounts to tissues [1,2,3]. In our case, 2.5 atmospheres were utilized for 90 min.

The results acquired proved that improvement of hearing in patients with AAT is inversely proportional to the intensity of the damage. Thus, patients whose condition improved after the treatment had less severe hearing impairment before the start of the treatment in comparison with patients whose condition did not ameliorate after treatment—at 4000 Hz (at the significance level <0.05).

In terms of our results, it is possible to state, in addition, that hearing improvement depends on the age of the patient, thus patients who are statistically younger (average age of 34.4 years) see greater improvement in condition than older ones (average age of 44.4 years).

Lamm and Arnold demonstrated, on an animal model, the subsequent decrease of partial pressure in perilymph and the decrease of the amplitude of cochlear potentials during the first 24 min after AAT [4]. They, in turn, observed an immediate increase of these parameters, promptly after HBO2 therapy [4].

According to Reazee et al., the limit figure of the sound impulse causing AAT differs by various standards [19]. For instance, NATO set the safe sound threshold at 160 dB (for the army) [2]. The administration of safety and health protection at work considers 140 dB the safe limit of sound. In case of excessive exposure to noise exceeding this, the safe limit is further lowered [20,21]. Recently, an increased incidence of AAT has been a matter of discussion in connection with the use of Bren guns (a certain model of gun), which PTA displays damage prevalence between 3000–6000 Hz according to Mrena et al. [13] According to our data, the most severely damaged frequencies are 6000 Hz followed by 8000, 4000, and 2000 Hz respectively.

An interesting discovery in our dataset was found, as although we detected the success of the treatment lower in Group A to B (70 vs. 81%), there was a larger adjustment of hearing to normal in Group A over B (39 vs. 29%). Two possible influencing factors are considered—using new noisier Bren type guns and starting timely the treatment in Group A. For instance, Mardassi et al. [22] stated that improvement of hearing after treatment is 81% in a group of young soldiers (exposed to shots and explosions) (n = 64 ears). The hearing threshold improved, on average, by 14 dB. In our group, the amelioration after treatment was 8 dB. According to the Finnish study, it may be possible to prevent some AATs using careful planning of military exercises [12].

In addition, one of the most frequent symptoms in AAT is, excluding hypacusis and tinnitus. Its occurrence is caused by the disruption of functional compactness in hair cells and nerve fibers [2,4]. In the study by Jokitulppo et al., tinnitus after AAT occurred in more than 60%. Furthermore, the study states the exposure to noise correlates with subsequent tinnitus [12,23], which is evident in our group of patients, where the occurrence of tinnitus was similarly 58%.

Improvement of hearing depends on the age of the patient and several others additional comorbidities. The eventual presence of an endotympanic effusion must be evaluated and treated with a ventilation tube so that the auditory tube re-establishes its physiological function [24,25].

Van der Veen et al. in their study dealt with the clinical issue of how HBO2 therapy effects threshold figures of hearing in patients who suffered acute acoustic trauma [1]. They ascertained that the effect of HBO2 therapy on hearing thresholds in patients with hearing loss, caused by AAT, is not statistically significant. Thus, they recommend performing distinctly designed randomized controlled studies with a sufficient set of patients that could conclude HBO2’s therapeutically effect in the treatment of AAT [1].

In our set, there were 52% ears in total, damaged by AAT, exposed to HBO2 therapy, and a positive effect was detected in 77%. On this basis, it may be possible to state that HBO2 therapy has a role in the treatment of AAT. A similar conclusion was given in a paper by Lafère et al. [26]. Also, Oya et al. published data on HBO2 therapy: 26 of the 37 ears (70%) displayed improved hearing [27].

In our study, we did not compare identical groups of patients (with HBO2 and without HBO2), because HBO2 therapy is indicated only in the case when pharmacological treatment is ineffective. Therefore, in accordance with Van der Veen et al., we recommend further studies so that it’s conclusively possible to answer the question of the effectiveness of HBO therapy in AAT treatment [1].

## 5. Conclusions

In the Czech Republic, many patients with AAT are members of the armed forces and their trauma is often classified as an accident at work. Based on our study, we can confirm the positive effect of early initiated corticosteroid treatment and HBO2 therapy. The application of a treatment protocol, well-timed administration of corticosteroids and initiation of HBO2 (no later than 7 days after acoustic trauma) may help to improve hearing in a patient with AAT. Because many patients are military personnel who suffer from AAT after small arms fire, prevention and proper use of protective equipment are strongly recommended. Hyperbaric oxygen therapy shows to be an adjuvant option in AAT, unfortunately we did not get the same real evidence of effectiveness in recovery from concomitant tinnitus.

## Figures and Tables

**Table 1:** Success of AAT treatment, All AAT, Group A (soldiers), Group B (civilians person).

	All AAT		Group A		Group B		*p*-Value *
Total Number of Damaged Ears	n = 141		n = 83		n = 58		
Improved in total	111	79%	58	70%	47	81%	0.096
Restored to standard = after treatment normacusis	58	41%	32	39%	17	29%	0.170
Partially improved	53	38%	26	31%	30	52%	0.012
Not improved	30	21%	25	30%	11	19%	

* Fisher’s exact test.

**Table 2:** (**a**) Latency of the beginning of the treatment—the whole set (total AAT). (**b**) Latency of the beginning of the treatment—Group A (soldiers). (**c**) Latency of the beginning of the treatment—Group B (civilian persons).

(**a**)			
**Latency of the Beginning of the Treatment**	**Total AAT**	**Improved after Treatment**	***p*-Value**
	**n = 141 Ears**		
Within 24 h	n = 56; 40%	n = 54; 96%	<0.001
Within 7 days	n = 55; 39%	n = 41; 74%	
After 7 days	n = 30; 21%	n = 16; 53%	
(**b**)			
**Latency of the Beginning of the Treatment**	**Group A**	**Improved after Treatment**	***p*-Value**
	**n = 83 Ears**		
Within 24 h	n = 36; 43%	n = 35; 97%	<0.001
Within 7 days	n = 28; 34%	n = 22; 79%	
After 7 days	n = 19; 23%	n = 10; 53%	
(**c**)			
**Latency of the Beginning of the Treatment**	**Group B**	**Improved after Treatment**	***p*-Value**
	**n = 58 Ears**		
Within 24 h	n = 20; 34%	n = 19; 95%	0.017
Within 7 days	n = 27; 47%	n = 19; 70%	
After 7 days	n = 11; 19%	n = 6; 55%	

* Fisher’s exact probability test.

**Table 3:** Success of particular medical protocols—Group I (corticosteroids therapy without HBO2), Group II (corticosteroids therapy with HBO2) and Singled out group (p. o. vasodilatants).

Group I (corticosteroids without HBO2)	n = 61 ears
Improved	50–82%
Improved to normacusis (threshold of losses above 20 dB)	39–64%
Group II (corticosteroids with HBO2)	n = 73 ears
Improved	56–77%
Improved to normacusis (threshold of losses above 20 dB)	27–37%
Singled out group p. o. vasodilatants	n = 7 ears
Improved	6–86%
Improved to standard (threshold of losses above 20 dB)	2–29%

**Table 4:** Group I (corticosteroids therapy without HBO2) and Group II (corticosteroids therapy with HBO2) changes of threshold of hearing before and after the treatment.

Frequency	Group	N	Average (dB)	St. Deviation	*p*-Value *
125	I	61	1.48	4.117	0.984
	II	73	1.16	6.265	
250	I	61	1.39	3.180	0.121
	II	73	3.15	7.193	
500	I	61	1.23	3.248	0.007
	II	73	4.04	7.530	
1000	I	61	2.21	5.666	0.284
	II	73	3.63	8.261	
2000	I	61	3.11	5.490	0.043
	II	73	7.05	10.924	
4000	I	61	6.80	10.248	0.146
	II	73	10.41	12.984	
6000	I	61	9.18	16.985	0.767
	II	73	10.27	17.298	
8000	I	61	9.26	11.862	0.468
	II	73	8.56	16.083	

* Mann-Whitney test.

## Data Availability

Not applicable.
